# Identification and Characterization of Families That Are Positively Deviant for Childhood Obesity in a Latino Population: A Case-Control Study

**DOI:** 10.1155/2018/9285164

**Published:** 2018-06-19

**Authors:** Byron A. Foster, Christian A. Aquino, Sharol Mejia, Barbara J. Turner, Arvind Singhal

**Affiliations:** ^1^Departments of Dermatology and Pediatrics, Oregon Health & Science University, Portland, OR, USA; ^2^Center for Research to Advance Community Health, University of Texas Health San Antonio, San Antonio, TX, USA; ^3^School of Medicine, University of Texas Health San Antonio, San Antonio, TX, USA; ^4^Department of Communication, The University of Texas at El Paso, El Paso, TX, USA; ^5^Inland University of Applied Sciences, Elverum, Norway

## Abstract

**Background:**

Childhood obesity is a complex public health challenge that requires innovative, sustainable solutions. Positive deviance, inspired by the science of complexity, is an approach that examines what allows certain individuals to succeed despite being predicted to fail. This study is aimed at identifying and defining positive deviants for early childhood obesity.

**Methods:**

This case-control study used medical record data to identify Latino children aged 2–5 and classify them using their longitudinal weight change. Parents of children with trajectories toward a healthy weight from an obese weight (cases) and parents of children with stable obese weight trajectories over time (controls) were recruited. Mixed-methods analyses were used including a semistructured interview and quantitative surveys evaluating diet, physical activity, sleep, feeding practices, and self-efficacy. Qualitative description was applied to the qualitative data; quantitative data were analyzed using descriptive statistics and logistic regression modeling.

**Results:**

Of eligible Latino children identified from the overall data set (*n*=1,621), 257 (16%) had trajectories toward a healthy weight, and among these, 21 positively deviant cases completed the study with 23 matched controls. Positive deviant families were characterized by lower education, higher self-efficacy, and a more Mexican cultural orientation. Findings suggest that effective engagement of other caregivers and creating healthy food environments were important determinants of healthy weight outcomes.

**Conclusions:**

Positive deviants (cases) were distinct from controls in several parenting strategies such as creating healthy food environments and engaging caregivers. They had higher self-efficacy despite lower education. There were fewer differences in diet and physical activity than expected.

## 1. Introduction

As early childhood obesity strongly predicts adolescent and adult weight [1], effective interventions directed at this age group are needed to reduce lifetime risk of diabetes and cardiovascular disease. Low-income Latino children are at highest risk for obesity among racial-ethnic groups [2] and experience significant healthcare access disparities [3]. Prior interventions have demonstrated mixed results in Latino, preschool-aged children. Well-designed, theory-based interventions showed no effect on adiposity in Head Start [4, 5] or community settings [6]. However, other studies using either a home-based intervention targeting healthier routines [7] or a parenting skills intervention on nutrition have shown more promising results [8]. These mixed results highlight the need for further community-based research. Approaches that identify what is already working in low-income Latino families can inform the development of interventions for the larger community.

Positive deviance is an approach to solve complex problems such as childhood obesity that asks, theoretically, what is working in the community, particularly among the most disadvantaged ones [9, 10]. By identifying individuals who have found a way to achieve a desirable outcome in spite of adversity, and discovering their uncommon and replicable practices, the positive deviance approach presents an innovative, inside-out approach to solve complex problems [11–13]. In other words, there may be families at high risk for continued obesity who have successfully achieved a healthy weight and have used existing assets and resources to do so. Identifying their behaviors may yield lessons applicable for the rest of the community.

Applications of positive deviance theory to childhood malnutrition are well established [14, 15]; however, applications to address childhood obesity are very limited. Of the few studies published, their limitations include examination of cross-sectional data on weight [16] or the lack of control groups [17]. The current study addresses these limitations by identifying subjects through a longitudinal analysis of children's weight, and employing a case-control design, coupled with mixed-methods analysis to define positive deviants' characteristics and behaviors. We examined both known associations with obesity (feeding behaviors, physical activity, sleep, and screen time) along with a semistructured interview to elicit potentially novel behaviors.

## 2. Methods

### 2.1. Overall Design

This study identified children as positive deviants (cases) who had been obese and then were able to reduce their adiposity to a healthier weight. Children who remained persistently obese served as controls. Parents of the children classified as cases and controls were recruited to participate in this mixed-methods study that included a semistructured interview and a questionnaire on their lifestyles, eating habits, and physical activity.

### 2.2. Study Participants and Growth Trajectory Analysis

Medical record data from a large, safety-net healthcare provider in San Antonio, TX, were used to identify the weight trajectories. We focused on the most recent data from 2013 to 2015 (49,860 children) to examine weight-based trajectories and to minimize recall bias in our parental interviews. A data analyst with the health system pulled the growth chart data from the medical records using an HIPAA waiver obtained for this study.

Children were included in the growth trajectory analysis if they were obese (≥95th percentile for body mass index (BMI) per CDC guidelines) during 2–5 years, had at least three height and weight measurements over more than one year to determine a BMI *z*-score trajectory, were of Latino ethnicity, and lived in a San Antonio zip code.

A latent class linear mixed model with a linear link function and a diagonal variance-covariance structure was used to classify each child into a distinct group based on their BMI *z*-score trajectory [18]. Due to high variability in the BMI *z*-scores over time, a median filter (Tukey's Running Median Smoothing [19]) was used to reduce noise in the data. The variables used to model the repeated BMI *z*-scores were sex and age. Sex was treated as a fixed effect, while age varied by subjects (random effect) who entered the study at different times. The mean posterior probabilities of class membership were used to assess the classification accuracy, which in turn determined the number of classes allowed in the model.

### 2.3. Recruitment

**Figure 1:**
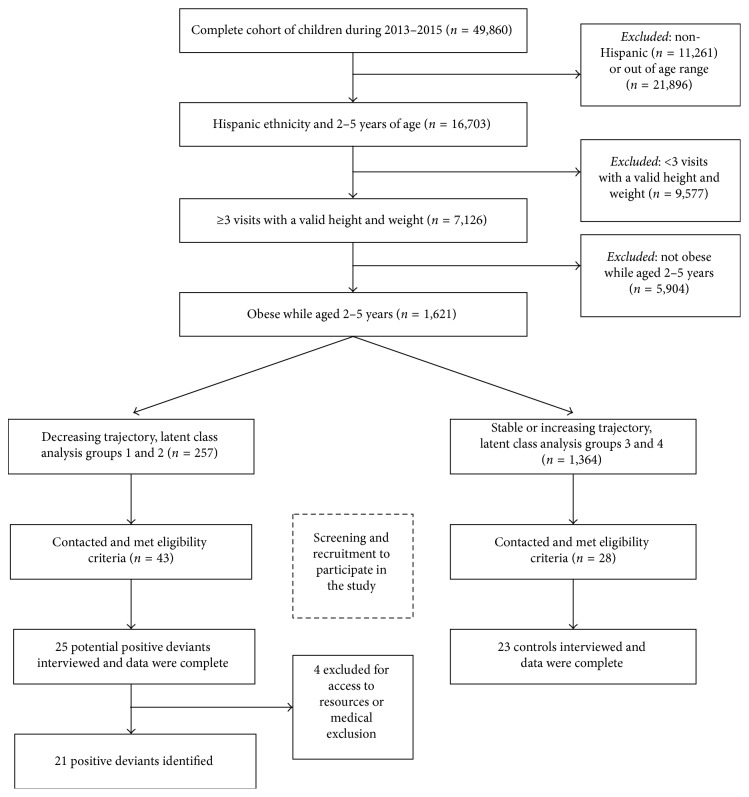
Identification of cases (positive deviants) and controls from a large, healthcare data set using inclusion and exclusion criteria as well as latent class trajectories.

After classification of children by weight trajectory, their parents were recruited to participate as either cases (positive deviants) or controls (Figure 1). Potential positive deviants had a negative BMI *z*-score trajectory over time (from an obese toward a healthy weight), were of Latino ethnicity, and with public insurance (based on their medical record). Controls had either a flat or an increasing BMI *z*-score trajectory by the latent class modeling and were matched by ethnicity and insurance status. Growth trajectory classes were visually confirmed on growth charts prior to recruitment. The sample size was determined by the qualitative data until thematic saturation was reached.

A letter, in English and Spanish, outlined the purpose and design of the study and was sent to all of the potential positive deviants. An option allowing recipients to return a postcard opting out of further communication was provided. An HIPAA waiver was obtained as part of the research protocol to do this. We recruited controls from the larger population of controls. A research assistant conducted follow-up phone calls with potential participants to determine if they met the eligibility criteria. Children were excluded from participation as either a case or a control if they had a diagnosis of intellectual disability or developmental delay, a seizure disorder, diabetes, cerebral palsy, a genetic problem, or if they were taking medication for attention deficit/hyperactivity disorder (these medications affect growth trajectories and physical activity).

### 2.4. Study Visit and Measures

Parents participated in a semistructured interview in either English or Spanish, followed by a quantitative assessment at either a clinical research center, a local library, or their home. All of the quantitative measures described below have been validated in Spanish except for the Comprehensive Feeding Practices Questionnaire [20]. Quantitative data were collected on REDCap [21].

### 2.5. Semistructured Interview

The semistructured interview was designed to elicit parents' opinions about the health of their child, including any changes purposely adopted or attempted related to their child and their child's health. Questions were grouped into general health and perceptions of health, feeding practices, physical activities, changes in weight, food purchasing and preparation, parent modeling, family rules and interactions, neighborhood and community resources, and school.

### 2.6. Demographics

The structure of the Behavioral Risk Factor Surveillance System Questionnaire [22] was used to gather demographic characteristics including parental sex, height, weight, race/ethnicity, household size, income, employment, and education.

### 2.7. Food Frequency Questionnaire

The Block Kids Food Screener (BKFS), developed by NutritionQuest (Berkeley, CA, USA) [23], was administered to parents to assess their child's dietary intake of nutrients and food groups.

### 2.8. Feeding Practices

The Comprehensive Feeding Practices Questionnaire (CFPQ) [20] consisting of 49 questions and 12 Likert subscales was administered. Parents self-reported the frequency and degree of agreement/disagreement with described practices and behaviors.

### 2.9. Screen Time, Sleep, and Outside Play

Participating parents were queried on their child's screen time using questions from the National Survey of Early Childhood Health [24]. Previously validated questions about their sleep habits [25] and outside play [26] were asked.

### 2.10. Food Security

To explore the relationship between food insecurity and obesity among low-income families, we asked questions to parents to assess their level of food insecurity using the US Department of Agriculture's (USDA) questions [27].

### 2.11. Self-Efficacy

To explore potential relationships between self-efficacy and effective weight maintenance, parents also completed the General Self-Efficacy Scale [28].

### 2.12. Acculturation

To gauge the differential diversity of acculturation among Latino children in San Antonio, the Acculturation Rating Scale for Mexican Americans-II (ARSMA-II) was administered [29]. This included two subscales on the degree of Anglo and/or Mexican Orientation and three subscales that examined the degree of marginalization.

### 2.13. Data Analysis

#### 2.13.1. Qualitative

Each semistructured interview was recorded and professionally transcribed in either English or Spanish, and all Spanish interviews were translated to English (Verbal Ink, Los Angeles, USA). Dedoose software was employed to facilitate the collaborative coding process. Each semistructured interview was coded independently by two authors using qualitative description, and differences were resolved through discussion. Qualitative description was used as the analytic method to stay “close” to data and be pragmatic. The purpose of qualitative coding was to capture events and experiences through rich descriptions and not to generate theory from data or serve a deep interpretive function [30, 31].

#### 2.13.2. Quantitative

Descriptive statistics were used to compare the groups by demographic characteristics and their responses to the administered surveys using SPSS 23.0 (IBM, USA). Logistic regression was performed to assess the strongest predictors of positive deviance status (dependent variable) with factors significant on bivariate analysis at *p* ≤ 0.10 included in the forward, stepwise regression analyses, using a probability of Wald statistics for variable elimination.

### 2.14. Ethics and Incentives

This study was approved by the Institutional Review Board of the University of Texas Health Science Center at San Antonio. The Bexar County Translational Advisory Board provided input at all stages of the study. All parents provided written informed consent to participate and received a $50 stipend for their participation.

## 3. Results

### 3.1. Identification of Positive Deviants

Of the 49,860 children in the data set from 2013 to 2015, there were 16,703 who were both Latino and 2–5 years of age (Figure 1). Of these, 57% (9,577) were excluded for reason of not having 3 or more visits spread over at least 12 months in order to assess a weight trajectory. Finally, 23% (1,621) of that sample was found to be obese (≥95th BMI percentile for age and sex) during the period and thus eligible for inclusion. From the latent class analysis, 16% (257) of children had a decreasing trajectory over time for their BMI *z*-score (groups 1 or 2) and 84% (1,364) had a stable or increasing trajectory (groups 3 or 4). Of the 257 children with a decreasing trajectory, we were able to contact and screen 43 children who were potentially positive deviants with 25 completing the study visit. Of the 1,364 controls, we screened them for eligibility on a rolling basis as we contacted them, with 23 controls completing the study, matched on ethnicity and insurance. The difference in the recruitment rate can be explained pragmatically: if a control needs to reschedule a visit, we moved onto the next control on the list. However, for positive deviants, given the limited number, we kept attempting to reschedule though that proved futile.

Since the screening did not fully evaluate the degree of access to resources (a core component to determine positive deviance), we reviewed the educational level and income data of the enrolled (potentially positively deviant) participants. Of the positive deviants interviewed, three had access to extra resources (either having a high income or being a medical provider) and one was identified as starting a medication for attention deficit disorder (something not identified at the time of screening). These participants were excluded from the final analysis.

### 3.2. Demographic Characteristics of Participants

The mean age of children enrolled was 71 months (SD = 15), 22/44 (50%) families reported an income of less than 25,000 USD per year, and 37/44 (84%) were on public insurance (Table 1). About half (24/44) of family members chose to complete the interview and surveys in English and the rest in Spanish. Six subjects reported having private insurance during the interview whose medical record had identified them as having public insurance. The positive deviants had a lower level of education than the controls, with most (69%) having less than a high school education compared with 39% of controls (*p*=0.02). As expected, the BMI *z*-score was lower at the time of enrollment in the positive deviance group compared with the controls (*p* < 0.001) (Table 1).

### 3.3. Feeding Practices

Using the Comprehensive Feeding Practices Questionnaire, positive deviants reported a higher level of agreement with practices related to restricting their child's diet to limit less healthy foods and sweets on a Likert scale from 1 to 5, with a mean score of 4.1 (SD = 1.0) versus a mean of 3.5 (SD = 1.0) for controls (*p*=0.06, *t*-test). There were no differences in the subscales of modeling, food as reward, encouraging balance and variety, the food environment, and restriction for weight control (Table 2).

### 3.4. Dietary Intake

After screening the food frequency data for quality, 43 children had valid food frequency data. Overall, the two groups were similar in their dietary intake overall with no major differences in nutrient or food group estimates at *p* < 0.05 (Table 2). The only observed difference at the *p* < 0.10 level between positive deviants and controls was lower mean amounts of total protein (*p*=0.09).

### 3.5. Physical Activity, Sleep, and Screen Time

Positive deviants reported higher outside physical activity time per week (*p*=0.09). There were no significant differences in reported screen time (data not shown) or sleep patterns between the groups (Table 2).

### 3.6. Self-Efficacy, Acculturation, and Food Security

Overall, a trend of higher overall self-efficacy was observed in the positive deviant group compared with controls (*p*=0.10) (Table 2). There were no differences in the three subscales of self-efficacy (initiative, effort, or persistence).

There was a different distribution of acculturation levels between the positive deviants and controls (*p*=0.01) (Table 2), with positive deviants having more of a Mexican orientation. There were no major differences seen in the marginalization subscales (data not shown).

Only 59% of the families had high food security (Table 2). There were no differences by group on food security status as assessed by the USDA measure.

### 3.7. Multivariable Analysis

For variables associated with being a positive deviant at *p* < 0.10 in the bivariate analysis, we examined the data for potential interactions between significant variables. Across all parents, self-efficacy was positively associated with education and Pearson's correlation coefficient (*r*) = 0.39 (*p* < 0.01). However, when examining positive deviants and controls separately, this association was only found to be true for controls with *r*=0.60 (*p* < 0.01). For positive deviants, there was no significant correlation between general self-efficacy assessed with the GSES and education (*r*=0.17; *p*=0.46).

Given that education was associated with being a positive deviant and in an unexpected direction, we further examined the subset of parents with low education (less than high school) for differences between positive deviants and controls. Only looking at those with less than a high school education, there was a difference in self-efficacy between positive deviants (mean GSES initiative scale = 4.2 (SD = 0.7)) and controls (mean GSES initiative scale = 3.3 (SD = 1.3); *p*=0.04).

Stepwise logistic regression model building for prediction of positive deviance status yielded a final model with variables for self-efficacy and education level with an overall classification accuracy of 74% and a Nagelkerke's R^2^ of 0.37. The continuous variable self-efficacy had an estimated OR of 5.0 (95% CI: 1.2–21.2; *p*=0.03). The education level less than high school compared with college or more had an estimated OR of 20.1 (95% CI: 2.7–149.6; *p*=0.003) and high school compared with college or more had an estimated OR of 17.0 (95% CI: 1.8–163.7; *p*=0.01).

### 3.8. Qualitative Analysis

There were five different thematic foci that emerged from the qualitative data analysis that distinguished positive deviants (Table 3). First, positive deviants created a healthy food environment. They did not favor having unhealthy foods in the house and purposely took healthy foods out of the house with them on outings in order to have a healthy option available. Second, positive deviants displayed message consistency: they believed that their child knew what foods they could or could not eat for snacks. Third, positive deviants expressed greater self-efficacy in making changes, for instance, being proactive to overcome their child's picky eating habits in contrast to let it be an ongoing challenge (picky eating was more common in controls). Fourth, positive deviants made incremental changes; for instance, they were more likely to reduce unhealthy snacks rather than trying to eliminate them completely, or were more likely to gradually increase time spent on physical activity rather than abruptly introduce a new rigorous physical activity. Finally, positive deviants successfully engaged the whole family in implementing changes, including engaging the grandparents, and also found opportunities for the whole family to be engaged in a collective physical activity.

What were the notable similarities across groups? Both positive deviants and controls tended to implement rules and limits for the entire family rather than for the individual child. Fathers tended to be more lenient when it came to eating practices in both groups. Controls often described the challenge of other lenient caregivers (fathers and grandparents) feeding their child whatever they wanted, whereas positive deviants largely described this as a challenge they overcame. There were no differences noted in eating together as a family (commonly described), perceptions of good and bad foods, using food as a reward, dealing with food insecurity, grocery shopping, food preparation, serving size, utilization of nutritional assistance, or eating out. The qualitative descriptions of physical activity were remarkably similar between positive deviants and controls in types of activity, frequency, and degree of parent involvement. No differences in the qualitative data were seen in descriptions of use of community resources, food security, health perceptions, parent modeling, or motivations (family members with diabetes being the most common).

Parents were specifically asked about goals or metrics they set related to their children's health and weight. There were no differences in the description of the goal between the groups. The most common goals were age-appropriate clothing sizes, less bullying at school, decreased weight (total body weight), and specific changes in diet patterns (e.g., less-fried foods). Notably, only positive deviants mentioned using doctor visits to track weight.

## 4. Discussion

This study is novel in utilizing a large healthcare data set to screen growth charts for potential positive deviants and including a set of controls. Furthermore, an in-depth, mixed-methods approach was employed to compare groups. Five primary qualitative themes distinguished positive deviants from the controls. Self-efficacy was identified as a quantitative predictor of positive deviance, despite the positive deviants having a lower level of educational achievement.

The creation of a healthy food environment emerged as a difference between positive deviants and controls in the qualitative data. Notably, there was no difference in parental self-rating of their creation of a healthy food environment on the CFPQ subscale with an overall mean of 4.2 (SD = 0.7) (*p*=0.83), perhaps a function of the social desirability bias. Although an association between the home food environment and early childhood obesity has been known [32], only recently have interventions specifically targeted home food environments. An intervention done in a relatively affluent, white population showed a significant reduction in adiposity [33], while an intervention done in a primarily Latino population showed no effect on the home food environment [34]. Family-based interventions that emphasize changes to the home environment have been effective in older age groups [35, 36].

Household-wide rule changes and message consistency were noted in another positive deviance investigation into childhood obesity [17]. In the current study, controls also attempted to implement household-wide rule changes, whereas consistency was in the domain of positive deviants. In the controls, there were multiple examples of parents reporting making household-wide changes to rules and then later describing the different exceptions to those rules. It is notable that the few interventions shown to be effective in this demographic have targeted household routines or emphasized consistency [7, 8].

We identified more restriction related to unhealthy foods but not restriction related to weight status among positive deviants. Restrictive feeding has been hypothesized to contribute to later obesity via the proposed sequelae of overconsumption of restricted, more palatable foods when the restrictions are relaxed [37], although longitudinal data have not confirmed that association [38]. The relationship between restrictive feedings may be in response to weight status [39], and interactions exist between the parenting style, child temperament, and restrictive feeding [40].

Successful engagement of other care providers was unique to positive deviants. Grandparents represented a common care provider, particularly for Latino families [41]. In this study, both grandparents and fathers were described as being more indulgent overall, consistent with prior findings [42]. The key difference in positive deviants was the mother's described ability to effectively change or reign in their indulgent behavior. Although this finding is unique to this study, one of the few other studies using this approach identified a similar construct of social support in positive deviants [43].

Self-efficacy emerged as a predictor of positive deviance in the quantitative data—a finding reinforced in the qualitative descriptions. This finding is consistent with previous studies: interventions designed to facilitate greater self-efficacy (i.e., motivational interviewing) are effective [44, 45]. The qualitative data suggested greater self-efficacy specific to picky eating in positive deviants. Recent longitudinal data on picky eating in early childhood and subsequent weight status have not shown a consistent relationship, although these studies were not done in a Latino population [46, 47]. A recent study in a low-income, mixed race, preschool population found no association between picky eating and obesity but did find an association with healthy eating patterns and picky eating [48]. The picky eating noted among controls in the current study was a significant source of frustration and may also reflect lower general self-efficacy.

The finding of lower education associated with positive deviance status was unexpected. The qualitative data seemed to indicate a pattern of positive deviants listening more to what the doctor told them about their child's weight status compared with controls who more often described the doctor's opinion as incorrect. Lower education has been associated with lower preference for shared decision-making and more paternalistic communication [49], and lower education has been associated with higher vaccine adherence [50]. This finding combined with high general self-efficacy may have contributed to the difference for the positive deviants. Remarkably, the GSES score for positive deviants with less than a high school education was similar to that of the controls with a college education.

We observed a significant association of positive deviance with a slight Mexican orientation on the acculturation scales. Among adults, it has been observed that lower acculturation to the dominant culture in the United States is associated with a healthier lifestyle [51]. However, in contrast to the adult data and our findings, prior studies in children have found an association between obesity and lower acculturation [52–54]. More recent adult data suggest that the observed association may be related to time in the United States and the community context rather than acculturation per se [55].

We observed a lower reported intake of protein among positive deviants compared with controls. In adult studies of dietary interventions for obesity, the data suggest that higher protein intake correlates with greater weight loss [56, 57]. However, our finding is consistent with cohort studies in early childhood examining macronutrient intake [58–60].

In general, positive deviance studies are well positioned to identify novel strategies to address complex problems by using qualitative methods to elicit previously unidentified themes. Notably, most of the studies applying positive deviance to malnutrition [15] are designed to apply findings directly to the community with a very short time between eliciting findings and implementation of the intervention. In contrast, the few studies using the positive deviance approach in obesity [16, 43, 61] have a longer time interval between eliciting findings and intervention in the community.

The vast majority of projects in the field of malnutrition pair up positive deviance with the hearth method of community engagement, teaching the local community about how they can use the findings from their own community in a community health effort. The work on obesity in the United States has been more research-focused. In this context, positive deviance may still provide insight into how to tailor interventions to the population and which components of an intervention are most relevant. Additionally, incorporating findings common across studies such as household-wide implementation of rules may prove to be beneficial.

### 4.1. Limitations

This study was not powered to detect a difference around the quantitative measures of physical activity, diet, sleep, or other factors. The fact that no major differences emerged in these categories could be either due to a lack of power, potentially social desirability bias in answering the questionnaires driving both responses upwards, or that no differences existed at the time of assessment. Also, parents' reported physical activity and total caloric intake do approximate recommendations in both groups, which may limit the external validity. Using the significance cutoff of *p* ≤ 0.10 allowed for identification of possible differences and use of the qualitative data to correlate across data types. However, this increases the possibility of type 1 error. Other limitations include the cross-sectional nature of the collected data and the potential for recall bias as the changes in behaviors or parenting were across time in growth chart trajectories. This study did not investigate data on other siblings' growth trajectories, or the parents' weight changes over the same period. The control group was matched by ethnicity and insurance status; the addition of neighborhood matching could have improved the matching process and controlled for other community-level changes. Although children of 2–5 years were eligible, using the weight trajectory definition of three or more measurements over a one-year period plus the time to conduct the analysis and do recruitment biased the sample toward an older population as seen by the mean age of five years.

## 5. Conclusion

Using electronic medical records to identify families of children successful in losing their weight identified several behavioral strategies that positively deviant parents employed, for instance, creating healthier food environments, providing consistent messages, effective engagement of caregivers and family members, and a proclivity to encourage incremental changes.

## Figures and Tables

**Table 1:** Baseline demographic characteristics of study children and parents.

	Controls (*n*=23)	Positive deviants (*n*=21)	All (*n*=44)	*p* value
Child age (months), mean (SD)^1^	71 (18)	69 (12)	71 (15)	0.75
Child sex (female), *n* (%)^2^	10 (44%)	10 (48%)	20 (46%)	0.51
Child BMI percentile, median (IQR)^3^	98.8 (96.9–99.3)	93.2 (82.8–96.5)	96.8 (91.6–99.2)	<0.01
Child BMI *z*-score, mean (SD)^1^	2.17 (0.60)	1.50 (0.72)	1.82 (0.74)	<0.01
Parental age (years), mean (SD)^1^	34 (7)	37 (11)	35 (9)	0.37
Interviewed parent sex (female), *n* (%)^2^	23 (100%)	20 (95%)	43 (98%)	0.48
Parent BMI, mean (SD)^1^	33 (7)	30 (7)	32 (7)	0.21
Preferred language (English), *n* (%)^2^	13 (57%)	11 (52%)	24 (55%)	1.0
Hispanic, *n* (%)^2^	23 (100%)	21 (100%)	44 (100%)	1.0
Household size, median (IQR)^3^	5 (4–6)	4 (3–5)	4 (3–5)	0.13

*Income,n(%)* ^2^				0.47
Less than $25,000	11 (48%)	11 (52%)	22 (50%)	
Less than $50,000	6 (26%)	7 (33%)	13 (30%)	
$50,000 or more	5 (22%)	1 (5%)	6 (14%)	
Not known/not sure	1 (4%)	2 (10%)	3 (7%)	

*Marital status,n(%)* ^2^				0.18
Married	14 (61%)	8 (38%)	22 (50%)	
Divorced/separated	1 (4%)	4 (19%)	5 (11%)	
Never married/member of an unmarried couple	8 (35%)	9 (43%)	17 (39%)	

*Education level,n(%)* ^2^				0.02
Less than high school	9 (39%)	14 (67%)	23 (52%)	
High school diploma or equivalent	3 (13%)	5 (24%)	8 (18%)	
Any college or college graduate	11 (48%)	2 (10%)	13 (30%)	

*Insurance,n(%)* ^2^				0.67
Private	4 (17%)	2 (10%)	6 (14%)	
Public	19 (83%)	18 (86%)	37 (84%)	
None	0	1 (5%)	1 (2%)	

^1^ *t*-test; ^2^chi-squared test; ^3^Mann–Whitney test.

**Table 2:** Parent-reported general self-efficacy, acculturation, food security, child physical activity, child sleep habits, and child dietary patterns between positive deviants and controls.

	Controls (*n*=23)	Positive deviants (*n*=21)	All (*n*=44)	*p* value
*General self-efficacy, mean (SD)*				
Overall score	4.24 (0.67)	4.41 (0.48)	4.32 (0.59)	0.10
Initiative score	3.95 (1.04)	4.43 (0.69)	4.18 (0.91)	0.17
Effort score	4.41 (1.07)	4.37 (0.65)	4.39 (0.89)	0.12
Persistence score	4.38 (0.71)	4.43 (0.75)	4.40 (0.72)	0.92

*Acculturation levels,n(%)*				0.01
Very Mexican oriented	8 (35%)	5 (25%)	13 (30%)	
Mexican oriented to approximately balanced bicultural	5 (22%)	9 (45%)	14 (33%)	
Slightly Anglo oriented bicultural	10 (44%)	2 (10%)	12 (28%)	
Strongly Anglo oriented	0	4 (20%)	4 (9%)	

*Food security,n(%)*				0.43
High food security	11 (48%)	15 (71%)	26 (59%)	
Marginal food security	6 (26%)	2 (10%)	8 (18%)	
Low food security	5 (22%)	3 (14%)	8 (18%)	

*Physical activity*				
Days playing outside, mean (SD)	5.2 (1.9)	6.1 (1.7)	5.6 (1.8)	0.11
Time playing outside per day (minutes), mean (SD)	81 (45)	106 (74)	93 (61)	0.18
Active play time (hours per week), mean (SD)	7.2 (5.4)	11.1 (9.3)	9.1 (7.7)	0.09
*Sleep*				
Sleep (hours per day), mean (SD)	9.9 (1.7)	10.0 (1.0)	10.0 (1.4)	0.90
Child naps (yes), *n* (%)	7 (30%)	8 (38%)	15 (34%)	0.75

*Dietary patterns* (*estimates shown as mean (SD) intake per day*)				
Fruit, including fruit juice (cups)	1.4 (0.7)	1.5 (0.9)	1.4 (0.8)	0.50
Vegetables (cups)	0.6 (0.4)	0.5 (0.3)	0.6 (0.4)	0.84
Potatoes, including French fries (cups)	0.2 (0.2)	0.2 (0.2)	0.2 (0.1)	0.60
Whole grains (ounces)	0.5 (0.3)	0.3 (0.3)	0.4 (0.3)	0.11
Saturated fat (grams)	16.1 (7.3)	13.5 (5.7)	15.0 (6.7)	0.23
Meat, poultry, and fish (ounces)	2.4 (1.5)	1.8 (1.4)	2.1 (1.5)	0.24
Dairy (cups)	1.5 (0.8)	1.4 (0.5)	1.5 (0.7)	0.44
Legumes (cups)	0.2 (0.3)	0.1 (0.1)	0.2 (0.2)	0.55
Sugar added to foods/drink (tsp)	4.9 (2.2)	5.8 (5.2)	5.3 (3.7)	0.47
Energy intake (kcal)	1141 (447)	992 (336)	1080 (407)	0.27
Protein (grams)	51 (22)	40 (15)	47 (20)	0.09
Fat (grams)	46 (20)	38 (17)	43 (19)	0.21
Carbohydrate (grams)	134 (49)	127 (42)	131 (46)	0.61
Fiber (grams)	12 (6)	10 (3)	11 (5)	0.41
Sugars occurring in foods, juice (grams)	65 (25)	72 (28)	68 (26)	0.43
Energy from sugary beverages (kcal)	25 (25)	40 (77)	31 (52)	0.38
Sugary beverages (servings)	0.2 (0.2)	0.2 (0.3)	0.2 (0.2)	0.77

*CFPQ scales*				
Environment (parents make healthy food available in the home)	4.3 (0.6)	4.2 (0.8)	4.2 (0.7)	0.83
Restriction for health (parents control the child's food intake to limit unhealthy foods)	3.5 (1.0)	4.1 (1.0)	3.8 (1.0)	0.06
Parents use food as reward	2.3 (0.9)	2.1 (0.8)	2.2 (0.9)	0.34
Modeling (parents demonstrate healthy eating)	4.2 (1.0)	4.4 (0.9)	4.3 (0.9)	0.60
Encourage balance and variety	4.3 (0.6)	4.6 (0.6)	4.4 (0.6)	0.18
Restriction for weight control (parents control intake to influence weight)	3.1 (0.9)	2.9 (1.0)	3.0 (1.0)	0.63

kcal = kilocalories; SD = standard deviation.

**Table 3:** Representative quotes for each of the five themes identified from the qualitative description analysis alongside representative quotes from controls where appropriate.

Positive deviants	Controls
*1. Creating healthy food environments*	
“… why am I going to have this at the house and her being tempting in eating it. I'd rather not have it and this way whenever she opens the refrigerator well she's not going to be tempted.” PD13	“They can't just freely go out, open up a bag of chips. They'll say, ‘Mom, can I open up these Doritos?' Then, I'll look to see what I have to make sure… and I'll be like, ‘All right, well, just get a little bit.'” C1
“That's why I always make sure that they have them in the fridge, because I'd rather them eat as much fruit as they want than have to worry about giving them junk…. they're at the bottom of the fridge drawer. He just opens it.” PD5	“…but he likes to steal candy. He'll come in the middle of the night. He'll go in the refrigerator, take the yogurts. He'll take the sweet bread we leave out at night on the table.” C12
“When we leave from here, I take a container with fruit for her. Sometimes–she really likes eating celery sticks. I take some celery for her.” PD14	“…he's really disciplined, he'll tell me. ‘Mommy, can I grab this?' ‘Yes.' And potato chips, we do buy potato chips, but I don't let them eat an entire bag.” C50

*2. Message consistency related to snacks*	
“She really knows not to eat junk food, which she can't eat it because we don't have it. When she goes over to her cousins, she doesn't eat there. She knows.” PD28	“I noticed that the kids eat dinner over there and I cook dinner of here. There's like a free for all kind of. Yeah, there's was structure for them but it was like if the girls were hungry you know we're going to eat this, we're going to eat that. Just whatever they wanted.” C4
“Every Sunday we go out for walks with our bikes and he walks with us, for about two hours.” PD2	

*3. Confidence in making changes*	
“I don't drink soda anymore either, so me and her... It was just something I wanted to do… it hasn't been hard at all.” PD23	“I would I like for her to eat veggies, but she doesn't... Yeah, she doesn't like them.” C6
“…Just the food part. We're still having challenges on that. Hopefully, we can overcome that and just look for ideas. I'm always on Pinterest. I look at ideas of other parents because I'm pretty sure I'm not the only one that has picky eaters.” PD2	“I stay away from certain foods, but I don't... I guess, I do. I stay away from the foods that I know that my doctor would say no to. As far as portions, I've gotten smaller, but that's about it.” C8

*4. Making incremental changes*	
“Go out with them to play. Also before we hardly went out–and if we do not go out at least we do something for a while in the yard, we do activities, run or play or bicycle or so.” PD15	“He always wanted the Debbie cakes and ice cream, he was always stuck on peanut butter and jelly and then I changed it.” C5
“She would eat like bag of chips too… It's rare now. It's maybe once or twice, she'll eat it during the week.” PD23	

*5. Engaging the entire family*	
“Well, I mean, I figure we're all eating the same thing, because if I make changes with one–then one is going to say, “Why are you guys eating that and the rest are not?” So, everyone is the same. We all eat the same thing.” PD12	“I'm not drinking no more soda, so she doesn't drink soda no more… She just drinks when she's with my mom or sister, like I said, or her dad.” C9
“I already prohibited him from bringing her candy. I told him, ‘You can bring her things that she can eat, but healthy things. Fruit–bring her fruit, any kind of fruit you want to. She loves fruit, apples, grapes.' I did struggle for a while to get him used to it, but it's been a long time since he's brought her that.” PD14	“Yeah, because he'll go and he'll ask my grandpa, “Can I have this?” My grandpa will give it to him, even though I've already told my son no. My grandpa doesn't know that so it just depends.” C13
